# The Mediterranean diet displays an immunomodulatory effect that correlates with beneficial changes in carotid atherosclerosis

**DOI:** 10.1093/cvr/cvaf027

**Published:** 2025-02-24

**Authors:** Ana Maria Ruiz-Leon, Miguel Camafort, Aleix Sala-Vila, Rosa Gilabert, Isabel Núñez, Sara Castro-Barquero, Montserrat Fitó, Rosa María Lamuela-Raventós, Xavier Pintó, Ana García-Arellano, Emilio Ros, Ramon Estruch, Rosa Casas

**Affiliations:** Department of Internal Medicine Hospital Clinic, Institut d’Investigacions Biomèdiques August Pi i Sunyer (IDIBAPS), University of Barcelona, 149 Rosellon Road, Barcelona 08036, Spain; Centro de Investigación Biomédica en Red Fisiopatología de la Obesidad y la Nutrición (CIBEROBN), Institute of Health Carlos III, 5 de Monforte de Lemos Avenue, Madrid 28029, Spain; Mediterranean Diet Foundation, 28 Johann Sebastian Bach Road, Barcelona 08021, Spain; Nutrition and Food Safety Research Institute (INSA-UB), University of Barcelona, 171 Prat de la Riba Avenue, Santa Coloma de Gramanet 08921, Spain; Department of Internal Medicine Hospital Clinic, Institut d’Investigacions Biomèdiques August Pi i Sunyer (IDIBAPS), University of Barcelona, 149 Rosellon Road, Barcelona 08036, Spain; Centro de Investigación Biomédica en Red Fisiopatología de la Obesidad y la Nutrición (CIBEROBN), Institute of Health Carlos III, 5 de Monforte de Lemos Avenue, Madrid 28029, Spain; Centro de Investigación Biomédica en Red Fisiopatología de la Obesidad y la Nutrición (CIBEROBN), Institute of Health Carlos III, 5 de Monforte de Lemos Avenue, Madrid 28029, Spain; Cardiovascular Risk and Nutrition, Hospital del Mar Research Institute (IMIM), 88 Doctor Aiguader Road, Barcelona 08003, Spain; Diagnostic Imaging Centre, IDIBAPS, Hospital Clínic, 149 Rosellon Road, Barcelona 08036, Spain; Diagnostic Imaging Centre, IDIBAPS, Hospital Clínic, 149 Rosellon Road, Barcelona 08036, Spain; Centro de Investigación Biomédica en Red Fisiopatología de la Obesidad y la Nutrición (CIBEROBN), Institute of Health Carlos III, 5 de Monforte de Lemos Avenue, Madrid 28029, Spain; Nutrition and Food Safety Research Institute (INSA-UB), University of Barcelona, 171 Prat de la Riba Avenue, Santa Coloma de Gramanet 08921, Spain; Department of Nutrition, Harvard TH Chan School of Public Health, 665 Huntington Avenue, Boston, MA 02115, USA; Centro de Investigación Biomédica en Red Fisiopatología de la Obesidad y la Nutrición (CIBEROBN), Institute of Health Carlos III, 5 de Monforte de Lemos Avenue, Madrid 28029, Spain; Cardiovascular Risk and Nutrition, Hospital del Mar Research Institute (IMIM), 88 Doctor Aiguader Road, Barcelona 08003, Spain; Centro de Investigación Biomédica en Red Fisiopatología de la Obesidad y la Nutrición (CIBEROBN), Institute of Health Carlos III, 5 de Monforte de Lemos Avenue, Madrid 28029, Spain; Nutrition and Food Safety Research Institute (INSA-UB), University of Barcelona, 171 Prat de la Riba Avenue, Santa Coloma de Gramanet 08921, Spain; Nutrition, Food Science and Gastronomy, University of Barcelona, 27-31 Joan XXIII Avenue, Barcelona 08028, Spain; Cardiovascular Risk Unit, Internal Medicine Department, Hospital Universitari de Bellvitge, de la Feixa Llarga Road, L’Hospitalet de Llobregat 08907, Spain; Bellvitge Biomedical Research Institute (IDIBELL), 199 Granvia de l'Hospitalet Avenue, L’Hospitalet de Llobregat, Barcelona 08908, Spain; Faculty of Medicine, Universitat de Barcelona UB, de la Feixa Llarga Road, L’Hospitalet de Llobregat, Barcelona 08007, Spain; Department of Preventive Medicine and Public Health, Campus Universitario, School of Medicine, University of Navarra, 1 Irunlarrea Road, Pamplona 31008, Spain; Department of Internal Medicine Hospital Clinic, Institut d’Investigacions Biomèdiques August Pi i Sunyer (IDIBAPS), University of Barcelona, 149 Rosellon Road, Barcelona 08036, Spain; Centro de Investigación Biomédica en Red Fisiopatología de la Obesidad y la Nutrición (CIBEROBN), Institute of Health Carlos III, 5 de Monforte de Lemos Avenue, Madrid 28029, Spain; Department of Internal Medicine Hospital Clinic, Institut d’Investigacions Biomèdiques August Pi i Sunyer (IDIBAPS), University of Barcelona, 149 Rosellon Road, Barcelona 08036, Spain; Centro de Investigación Biomédica en Red Fisiopatología de la Obesidad y la Nutrición (CIBEROBN), Institute of Health Carlos III, 5 de Monforte de Lemos Avenue, Madrid 28029, Spain; Nutrition and Food Safety Research Institute (INSA-UB), University of Barcelona, 171 Prat de la Riba Avenue, Santa Coloma de Gramanet 08921, Spain; Department of Internal Medicine Hospital Clinic, Institut d’Investigacions Biomèdiques August Pi i Sunyer (IDIBAPS), University of Barcelona, 149 Rosellon Road, Barcelona 08036, Spain; Centro de Investigación Biomédica en Red Fisiopatología de la Obesidad y la Nutrición (CIBEROBN), Institute of Health Carlos III, 5 de Monforte de Lemos Avenue, Madrid 28029, Spain; Nutrition and Food Safety Research Institute (INSA-UB), University of Barcelona, 171 Prat de la Riba Avenue, Santa Coloma de Gramanet 08921, Spain

**Keywords:** Mediterranean diet, Cardiovascular disease, Inflammation, Carotid intima media thickness, Atherosclerosis, Cytokines

The Mediterranean Diet (MeDiet), a healthy, plant-based dietary pattern, is recommended by international scientific organizations for its potential in cardiovascular disease (CVD) prevention.^1^ However, its underlying mechanisms remain incompletely understood.^2^ Increased carotid intima-media thickness (CIMT), plaque height and inflammation are recognized as indicators of cardiovascular risk.^3,4^ Results of previous studies indicate that following the MeDiet may slow CIMT and carotid plaque progression^5^ and improve vascular inflammation.^6^ However, to our knowledge, no published studies have assessed whether changes in inflammatory biomarkers resulting from long-term MeDiet intervention correlate with changes in CIMT and plaque height. We hypothesize that MeDiet’s beneficial impact on atherosclerosis—through improving CIMT and plaque height—correlates with its immunomodulatory effects on inflammation and plaque stability-related molecules (*Figure 1A*).

**Figure 1:**
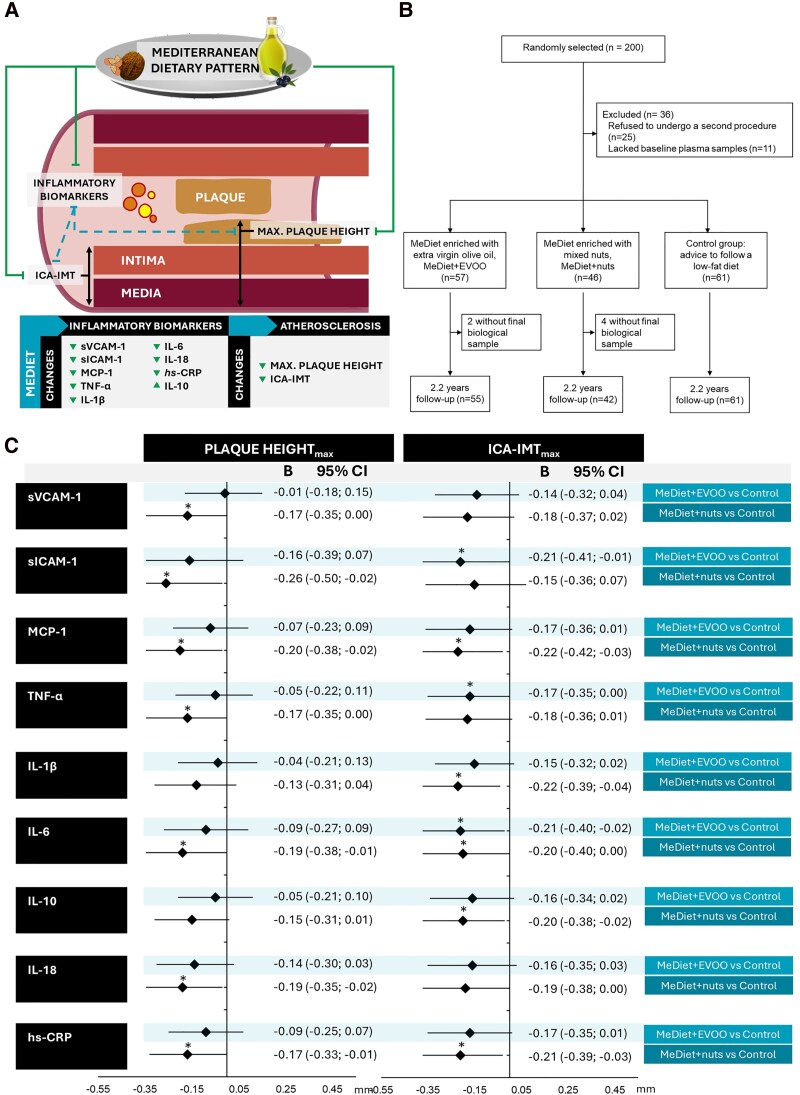
Results of an intervention study with a MeDiet supplemented with nuts or EVOO vs. a control diet (advice to follow a low-fat diet) after 2.2 years in an older Spanish population at high cardiovascular risk. (*A*) Graphical abstract: Schematic of the internal carotid artery showing inflammation markers, intima-media thickness (ICA-IMT) and maximum atheroma plaque height. Solid lines represent the known protective effect of the MeDiet on markers of inflammation, and on ICA-IMT and plaque height. Dashed lines represent the results of this study, which suggest that the MeDiet, especially when supplemented with nuts, shows an immunomodulatory effect correlating with beneficial changes in carotid atherosclerosis. (*B*) Flow diagram of study participants. (*C*) Adjusted forest plot of variation in maximum plaque height (mm) and maximum ICA-IMT (mm) in the MeDiet interventions with compared with the control after 2.2 years of study, related to changes in inflammation biomarkers (MeDiet + EVOO, *n* = 55; MeDiet + nuts, *n* = 42; control group, *n* = 61). General linear model approach to ANCOVA. Model 2 adjusted for sex, age at recruitment, baseline biomarker level, systolic and diastolic blood pressure, total-cholesterol and triglyceride concentrations, and medication use (hypoglycaemic drugs and statins). Black diamonds represent the standardized coefficient (*B*) together with the lines representing the 95% CI. *P*-values < 0.05 are marked with an asterisk (*). EVOO, extra-virgin olive oil; hs-CRP, high-sensitivity C-reactive protein; ICA-IMT, intima-media thickness of the internal carotid artery; sICAM-1, soluble intercellular adhesion molecule 1; sVCAM-1, soluble vascular cell adhesion molecule 1; IL-10, interleukin 10; IL-18, interleukin 18; IL-1β, interleukin 1 beta; IL-6, interleukin 6; MCP-1, monocyte chemoattractant protein 1; MeDiet, Mediterranean diet; TNF-α, tumour necrosis factor alpha.

Here, we report results from a secondary analysis of the PREDIMED (PREvención con DIeta MEDiterránea)^7^ study, a randomized, multicentre, parallel-group, single-blind controlled trial (RCT) conducted in Spain to assess MeDiet’s impact on primary prevention of CVD. Two hundred consecutive candidates from primary care centres linked to Clínic Hospital and Bellvitge Hospital (Barcelona, Spain) were screened between February 2008 and July 2009. Eligible participants, community-dwelling subjects, aged 55–80 for men and 60–80 for women, were free of CVD at baseline but at high risk. For complete protocol and eligibility criteria details see ISRCTN35739639 at www.isrctn.com. Institutional review boards at the two hospitals approved the study, which was conducted in accordance with the Declaration of Helsinki; all participants signed a written informed consent. Candidates were randomly assigned (1:1:1) to one of the three groups: MeDiet with extra virgin olive oil (EVOO, 1 L/week for the participants and their families), MeDiet with 30 g/day mixed nuts (walnuts, almonds, and hazelnuts), or control diet (advice to follow a low-fat diet). No energy restriction or physical activity was indicated for any group. Randomization was performed using a computer-generated random-number sequence. The primary outcome of this sub-study was the association between changes in plasma inflammation biomarkers and changes in carotid atherosclerosis (maximum CIMT and maximum plaque height) after intervention. Plasma inflammation and plaque stability-related molecules were assessed using the Luminex® system, and high-sensitivity C-reactive protein (hs-CRP) was determined by a standard enzyme-linked immunosorbent assay at baseline and was repeated at Years 1 and 3. A standardized ultrasonography imaging protocol was used to measure CIMT at the common carotid artery 1 cm pre-bifurcation (CCA-IMT), bifurcation (BIF-IMT), and internal carotid artery 1 cm after the flow divider (ICA-IMT), along with plaque height measurements^8^ at baseline and was repeated at Year 2. For each participant, the plasma biomarker measurements closest to the second ultrasound were used, reflecting changes after an average of 2.2 years of intervention (range: 1.6–3.1 years). Repeated-measures analysis of variance was used to compare changes in diet, adiposity, cardiovascular risk factors, and inflammation biomarkers, adjusting for potential confounding variables: age, sex, use of oral hypoglycaemic agents and lipid-lowering agents (statins), and baseline total-cholesterol and triglyceride levels, and systolic and diastolic BP. General Linear Model approach to analysis of covariance (ANCOVA) assessed associations between inflammation biomarkers changes in both MeDiet groups (fixed factors) compared with the control group on mean and maximum CCA-IMT, BIF-IMT, ICA-IMT, and maximum plaque height after an average of 2.2 years (dependent variables), using the ultrasonographic baseline measurements as covariates and others as additional covariates. Model 1 was unadjusted; Model 2 was adjusted for sex, age, baseline biomarker levels, systolic and diastolic BP, total-cholesterol and triglyceride concentrations, and medication use as oral hypoglycaemic drugs and statins.

As 25 participants declined a second ultrasound and baseline and follow-up plasma samples were missing for 11 and 6 participants, respectively, complete data were available for 158 participants (53% female) after a median of 2.2 years’ intervention (*Figure 1B*). The mean baseline age was 66.2 ± 5.9 years, with a mean CCA-IMT_max_ of 0.98 ± 0.27 mm and a mean plaque height_max_ of 1.86 ± 0.85 mm. All participants had similar clinical, dietary, and physical activity characteristics at baseline.

After 2.2 years of intervention, adherence to the MeDiet significantly increased in MeDiet groups compared with the control diet group, while physical activity was maintained in all groups. Participants in the MeDiet + EVOO group showed a significant increase in IL-10 levels, while those in the MeDiet + nuts group had a significant reduction in MCP-1. In contrast, participants in the control group displayed significant increases in the plasma concentrations of sVCAM-1, sICAM-1, TNF-α, IL-1β, IL-6, IL-18, and hs-CRP. A trend towards between-group differences in sICAM-1 and hs-CRP was observed in favour of both MeDiet arms (*P* = 0.05 and *P* = 0.06, respectively). *Figure 1C* shows the differences in maximum plaque height or ICA-IMT in the MeDiet interventions vs. the control diet after 2.2 years. The non-standardized coefficient (*B*) indicates the differences in maximum plaque height or ICA-IMT in the MeDiet interventions compared with the control diet, presented with its 95% confidence interval (95% CI). In the multivariable-adjusted Model 2, participants with the same maximum plaque height at baseline experienced a significantly greater reduction in plaque height_max_ (range: 0.17–0.26 mm) associated with plasma changes in sVCAM-1, sICAM-1, MCP-1, TNF-α, IL-6, IL-18, and hs-CRP after the MeDiet + nuts intervention compared with the control. Likewise, participants with the same ICA-IMT_max_ at baseline experienced a significantly greater reduction in the ICA-IMT_max_ (range: 0.20–0.22 mm), correlated with changes in plasma levels of MCP-1, IL-1β, IL-6, hs-CRP, and IL-10 after the MeDiet + nuts intervention compared with the control. ICA-IMT_max_ was also reduced by 0.17–0.21 mm, associated with changes in plasma levels of sICAM-1, TNF-α, and IL-6 after the MeDiet + EVOO intervention compared with the control. Mean CCA-IMT also showed a greater reduction [−0.12 mm (−0.24 to −0.001 mm)] associated with changes in plasma levels of MCP-1 after the MeDiet + nuts intervention compared with the control (data are not shown). No changes were observed in CCA-IMT_max_, maximum BIF-IMT_max_, mean ICA-IMT, or mean BIF-IMT.

The results of our study suggest that changes in ultrasound-assessed CIMT and plaque are associated with changes in concentrations of inflammation biomarkers ensuing from a MeDiet enriched with two of its main foods, nuts and EVOO. These findings may indicate that the immunomodulatory and anti-inflammatory properties of the MeDiet, which are linked to a reduced progression of atherosclerosis, might be responsible for the demonstrated effect in reducing the incidence of CVD observed in RCTs.^7,9^ The MeDiet’s effect to induce a reduced inflammatory status could be attributed to its richness in beneficial nutrients, such as dietary fibre, unsaturated fatty acids, and bioactive compounds, such as carotenoids and (poly)phenols, which may play an anti-atherosclerotic role.^2,10^

However, several limitations of this study should be noted, including a small sample size and the lack of a pre-specified sample size, as at the time of the ultrasound scans there were no published data on the maximum CIMT/plaque height progression to make assumptions for power calculations; nonetheless, the sample size of this exploratory study was sufficient to find evidence of an association between primary outcomes. Moreover, although these data support the link between the MeDiet’s beneficial effects on inflammation and atherosclerosis, the results show correlations between changes in variables after dietary intervention, which prevents conclusions of a clear cause-effect relationship being drawn. Additionally, our population comprised older individuals at high CVD risk living in a Mediterranean country, limiting generalization to other populations. The study also has several strengths, including its basis in the results of a RCT, real-life conditions with home-prepared foods, and close participant monitoring.

In conclusion, this study suggests that the MeDiet, especially when supplemented with nuts, displays an immunomodulatory effect that correlates with beneficial changes in carotid atherosclerosis. These findings propose a hypothesis for a new mechanistic explanation of how MeDiets may provide cardiovascular protection, warranting future research to validate this hypothesis.

## Authors’ contributions

M.C., R.E., and R.C.: study conception and design; A.M.R.-L., R.G., I.N., and R.C.: laboratory and clinical data; A.M.R.-L., M.C., S.C.-B., E.R., R.E., and R.C.: analysis and interpretation of the data; A.M.R.-L., M.C., R.E., and R.C.: draft of the article; A.M.R.-L., M.C., S.C.-B., A.S.-V., M.F., R.M.L.-R., X.P., A.G.-A., E.R., R.E., and R.C.: critical revision and final approval. A.M.R.-L., M.C., E.R., R.E., and R.C. wrote the paper. R.E. and R.C. had primary responsibility for the final content. All the authors have read and approved the final manuscript.

## Data Availability

The data underlying this article will be shared on reasonable request to the corresponding author.

## References

[1] Visseren FLJ, Mach F, Smulders YM, Carballo D, Koskinas KC, Bäck M, Benetos A, Biffi A, Boavida J-M, Capodanno D, Cosyns B, Crawford C, Davos CH, Desormais I, Di Angelantonio E, Franco OH, Halvorsen S, Hobbs FDR, Hollander M, Jankowska EA, Michal M, Sacco S, Sattar N, Tokgozoglu L, Tonstad S, Tsioufis KP, van Dis I, van Gelder IC, Wanner C, Williams B, De Backer G, Regitz-Zagrosek V, Aamodt AH, Abdelhamid M, Aboyans V, Albus C, Asteggiano R, Bäck M, Borger MA, Brotons C, Čelutkienė J, Cifkova R, Cikes M, Cosentino F, Dagres N, De Backer T, De Bacquer D, Delgado V, Den Ruijter H, Dendale P, Drexel H, Falk V, Fauchier L, Ference BA, Ferrières J, Ferrini M, Fisher M, Fliser D, Fras Z, Gaita D, Giampaoli S, Gielen S, Graham I, Jennings C, Jorgensen T, Kautzky-Willer A, Kavousi M, Koenig W, Konradi A, Kotecha D, Landmesser U, Lettino M, Lewis BS, Linhart A, Løchen M-L, Makrilakis K, Mancia G, Marques-Vidal P, McEvoy JW, McGreavy P, Merkely B, Neubeck L, Nielsen JC, Perk J, Petersen SE, Petronio AS, Piepoli M, Pogosova NG, Prescott EIB, Ray KK, Reiner Z, Richter DJ, Rydén L, Shlyakhto E, Sitges M, Sousa-Uva M, Sudano I, Tiberi M, Touyz RM, Ungar A, Verschuren WMM, Wiklund O, Wood D, Zamorano JL, Smulders YM, Carballo D, Koskinas KC, Bäck M, Benetos A, Biffi A, Boavida J-M, Capodanno D, Cosyns B, Crawford CA, Davos CH, Desormais I, Di Angelantonio E, Franco Duran OH, Halvorsen S, Richard Hobbs FD, Hollander M, Jankowska EA, Michal M, Sacco S, Sattar N, Tokgozoglu L, Tonstad S, Tsioufis KP, van Dis I, van Gelder IC, Wanner C, Williams B. 2021 ESC guidelines on cardiovascular disease prevention in clinical practice. Eur Heart J 2021;42:3227–3337. 34458905 10.1093/eurheartj/ehab484

[2] Violi F, Pastori D, Pignatelli P, Carnevale R. Nutrition, thrombosis, and cardiovascular disease. Circ Res 2020;126:1415–1442. 32379574 10.1161/CIRCRESAHA.120.315892

[3] Willeit P, Tschiderer L, Allara E, Reuber K, Seekircher L, Gao L, Liao X, Lonn E, Gerstein HC, Yusuf S, Brouwers FP, Asselbergs FW, van Gilst W, Anderssen SA, Grobbee DE, Kastelein JJP, Visseren FLJ, Ntaios G, Hatzitolios AI, Savopoulos C, Nieuwkerk PT, Stroes E, Walters M, Higgins P, Dawson J, Gresele P, Guglielmini G, Migliacci R, Ezhov M, Safarova M, Balakhonova T, Sato E, Amaha M, Nakamura T, Kapellas K, Jamieson LM, Skilton M, Blumenthal JA, Hinderliter A, Sherwood A, Smith PJ, van Agtmael MA, Reiss P, van Vonderen MGA, Kiechl S, Klingenschmid G, Sitzer M, Stehouwer CDA, Uthoff H, Zou Z-Y, Cunha AR, Neves MF, Witham MD, Park H-W, Lee M-S, Bae J-H, Bernal E, Wachtell K, Kjeldsen SE, Olsen MH, Preiss D, Sattar N, Beishuizen E, Huisman MV, Espeland MA, Schmidt C, Agewall S, Ok E, Aşçi G, de Groot E, Grooteman MPC, Blankestijn PJ, Bots ML, Sweeting MJ, Thompson SG, Lorenz MW. Carotid intima-media thickness progression as surrogate marker for cardiovascular risk. Circulation 2020;142:621–642. 32546049 10.1161/CIRCULATIONAHA.120.046361 PMC7115957

[4] Saba L, Saam T, Jäger HR, Yuan C, Hatsukami TS, Saloner D, Wasserman BA, Bonati LH, Wintermark M. Imaging biomarkers of vulnerable carotid plaques for stroke risk prediction and their potential clinical implications. Lancet Neurol 2019;18:559–572. 30954372 10.1016/S1474-4422(19)30035-3

[5] Sala-Vila A, Romero-Mamani ES, Gilabert R, Núñez I, De La Torre R, Corella D, Ruiz-Gutiérrez V, López-Sabater MC, Pintó X, Rekondo J, Martínez-González MÁ, Estruch R, Ros E. Changes in ultrasound-assessed carotid intima-media thickness and plaque with a Mediterranean diet: a substudy of the PREDIMED trial. Arterioscler Thromb Vasc Biol 2014;34:439–445. 24285581 10.1161/ATVBAHA.113.302327

[6] Casas R, Urpi-Sardà M, Sacanella E, Arranz S, Corella D, Castañer O, Lamuela-Raventós R-M, Salas-Salvadó J, Lapetra J, Portillo MP, Estruch R. Anti-inflammatory effects of the Mediterranean diet in the early and late stages of atheroma plaque development. Mediators Inflamm 2017;2017:1–12. 10.1155/2017/3674390 PMC5412172 28484308

[7] Estruch R, Ros E, Salas-Salvadó J, Covas M-I, Corella D, Arós F, Gómez-Gracia E, Ruiz-Gutiérrez V, Fiol M, Lapetra J, Lamuela-Raventos RM, Serra-Majem L, Pintó X, Basora J, Muñoz MA, Sorlí JV, Martínez JA, Fitó M, Gea A, Hernán MA, Martínez-González MA. Primary prevention of cardiovascular disease with a Mediterranean diet supplemented with extra-virgin olive oil or nuts. N Engl J Med 2018;378:e34. 29897866 10.1056/NEJMoa1800389

[8] Junyent M, Cofán M, Núñez I, Gilabert R, Zambón D, Ros E. Influence of HDL cholesterol on preclinical carotid atherosclerosis in familial hypercholesterolemia. Arterioscler Thromb Vasc Biol 2006;26:1107–1113. 16556855 10.1161/01.ATV.0000218507.95149.42

[9] Delgado-Lista J, Alcala-Diaz JF, Torres-Peña JD, Quintana-Navarro GM, Fuentes F, Garcia-Rios A, Ortiz-Morales AM, Gonzalez-Requero AI, Perez-Caballero AI, Yubero-Serrano EM, Rangel-Zuñiga OA, Camargo A, Rodriguez-Cantalejo F, Lopez-Segura F, Badimon L, Ordovas JM, Perez-Jimenez F, Perez-Martinez P, Lopez-Miranda J, Almaden Peña Y, Aranda E, Arenas de Larriva AP, Badimon JJ, Blanco-Molina A, Blanco-Rojo R, Bolivar-Muñoz J, Caballero-Villarraso J, Chica J, Corina A, Criado-Garcia J, Cruz-Teno C, Daponte-Codina A, de Teresa Galvan E, Delgado-Casado N, Estruch R, Fernandez JM, Fernandez-Gandara C, Fuentes-Jimenez F, Garcia-Carpintero Fernandez-Pacheco S, Gomez-Delgado F, Gomez-Garduño A, Gomez-Luna P, Gomez-Luna MJ, Gonzalez-Guardia L, Gonzalez-Requero AI, Gutierrez-Mariscal FM, Haro-Mariscal CM, Jimenez-Lucena R, Jimenez-Morales AI, Leon-Acuña A, Marin-Hinojosa C, Meneses Alvarez ME, Mesa-Luna D, Moya-Garrido MN, Muñoz-Carvajal I, Navarro-Martos V, Ochoa JJ, Ortiz-Minuesa JA, Pan M, Peña-Orihuela P, Perez-Corral I, Pi-Sunyer FX, Ramirez-Lara I, Rodriguez-Artalejo F, Romero MA, Roncero-Ramos I, Ruano-Ruiz JA, Ruiz de Castroviejo J, Sanchez-Villegas P, Suarez de Lezo J, Suarez de Lezo J, Vals-Delgado C, Valverde R, Visioli F. Long-term secondary prevention of cardiovascular disease with a Mediterranean diet and a low-fat diet (CORDIOPREV): a randomised controlled trial. Lancet 2022;399:1876–1885. 35525255 10.1016/S0140-6736(22)00122-2

[10] Koelman L, Egea Rodrigues C, Aleksandrova K. Effects of dietary patterns on biomarkers of inflammation and immune responses: a systematic review and meta-analysis of randomized controlled trials. Adv Nutr 2022;13:101–115. 34607347 10.1093/advances/nmab086 PMC8803482

